# Functional alterations of the magnocellular subdivision of the visual sensory thalamus in autism

**DOI:** 10.1073/pnas.2413409121

**Published:** 2024-11-11

**Authors:** Stefanie Schelinski, Louise Kauffmann, Alejandro Tabas, Christa Müller-Axt, Katharina von Kriegstein

**Affiliations:** ^a^Chair of Cognitive and Clinical Neuroscience, Faculty of Psychology, Dresden University of Technology, Dresden 01187, Germany; ^b^Max Planck Institute for Human Cognitive and Brain Sciences, Leipzig 04303, Germany; ^c^Laboratoire de Psychologie et Neurocognition, Université Grenoble Alpes, Grenoble 38000, France; ^d^Basque Center on Cognition, Brain and Language, San Sebastian 20009, Spain; ^e^Ikerbasque, Basque Foundation for Science, Bilbao 48009, Spain

**Keywords:** magnocellular, lateral geniculate nucleus, 7T-fMRI, Autism, visual motion

## Abstract

The long-standing hypothesis that autism is linked to changes in the visual magnocellular system of the human brain has never been directly examined due to technological constraints. Here, we used a recently developed 7-Tesla functional MRI (fMRI) approach to investigate this hypothesis within the visual sensory thalamus (lateral geniculate nucleus, LGN). The LGN is a crucial component of the primary visual pathway. It is particularly suited to investigate the magnocellular visual system, because within the LGN, the magnocellular (mLGN) uniquely segregates from the parvocellular (pLGN) system. Our results revealed diminished mLGN blood-oxygenation-level-dependent (BOLD) responses in the autism group compared to controls. pLGN responses were comparable across groups. The mLGN alterations were observed specifically for stimuli optimized for mLGN function, i.e., visual displays with low spatial frequency and high temporal flicker frequency. The results confirm the long-standing hypothesis of magnocellular visual system alterations in autism. They substantiate the emerging perspective that sensory processing variations are part of autism symptomatology.

Autism is a pervasive neurodevelopmental condition characterized by social interactive and communicative symptoms, repetitive behaviors, and restricted interests (1). It affects ~1% of people world-wide and often results in reduced social and economic opportunities (2). The mechanisms underlying the condition are controversially discussed and range from cognitive/emotional theories to sensory theories (2, 3). One long-standing hypothesis—based on behavioral findings and theoretical considerations—is that autism is associated with a visual magnocellular dysfunction (4, 5). The visual magnocellular system is specialized for processing rapid visual changes at large spatial scales. Conversely, the parvocellular system is specialized for small spatial scales and color processing (6). Studying these two systems separately in humans in vivo was for a long time impossible. Here, we used a 7-Tesla fMRI approach that allows imaging the visual magnocellular and parvocellular system in the primary visual thalamus, the lateral geniculate nucleus (LGN) based on an in-plane spatial resolution of 1.25 mm isotropic (M/P experiment). We included autistic (autism group) and nonautistic (control group) adults. Based on magnocellular theories of autism (4, 5), we hypothesized that the autism group would be characterized by dysfunction in the magnocellular (mLGN) but not the parvocellular (pLGN) subdivision of the LGN (6).

## Results

### Similar Overall Responses.

**Fig. 1.**
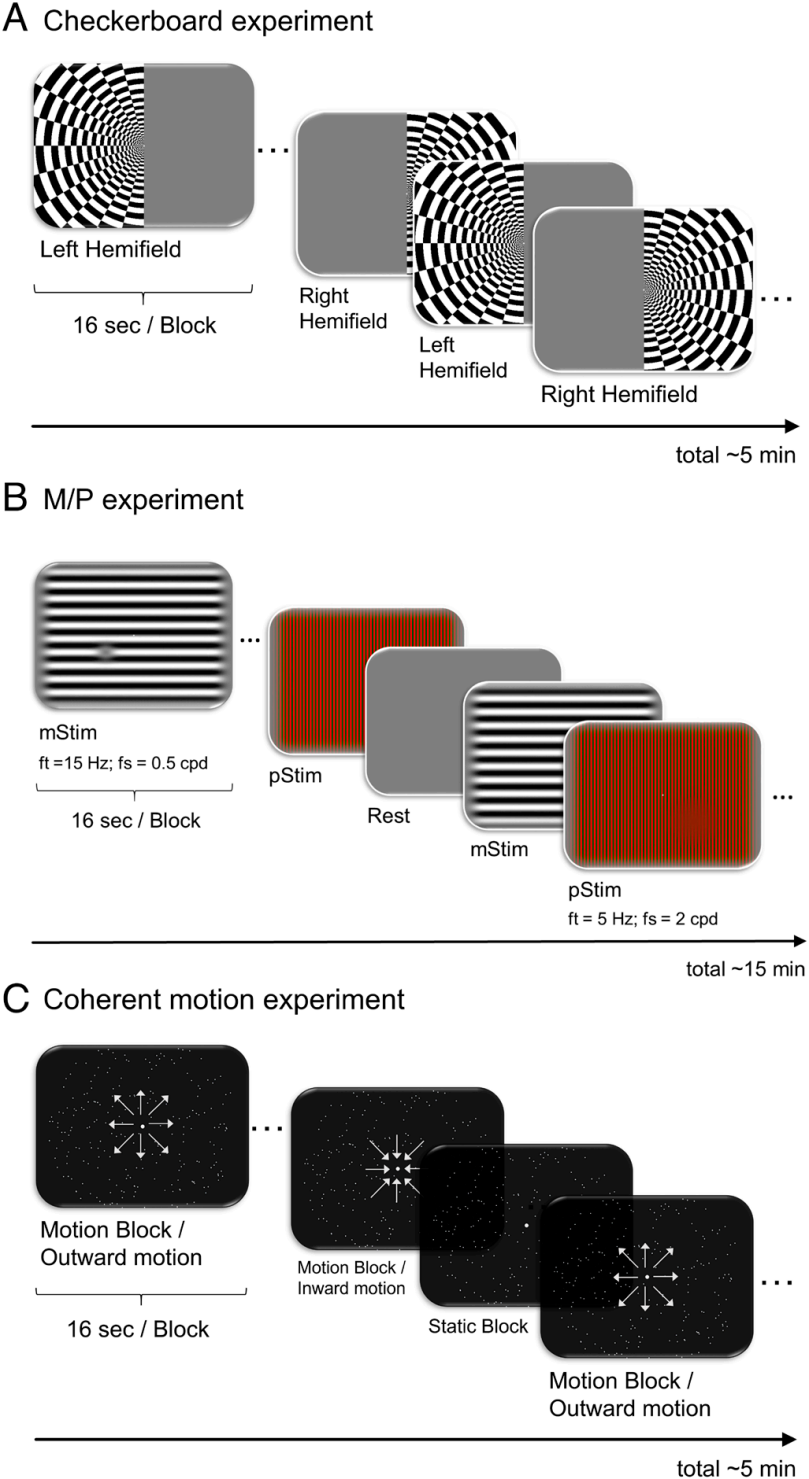
Experimental design and example trials. (*A*) Checkerboard experiment. Flickering checkerboards (100% contrast, 4 Hz contrast polarity reversal) were presented either on the left or the right half of the screen (Right Hemifield and Left Hemifield condition) alternating in a block design. (*B*) M/P experiment. M- and P-stimuli were both sinusoidal gratings with sinusoidal counterphase flicker. M-stimuli (mStim condition) were 100% luminance contrast black-white gratings with a spatial frequency (fs) of 0.5 cpd and a flicker frequency (ft) of 15 Hz. P-stimuli (pStim condition) were low-luminance, high color-contrast red-green gratings with a spatial frequency of 2 cpd and a flicker frequency of 5 Hz. Blocks of mStim, pStim, and a blank screen alternated. (*C*) Coherent motion experiment. Stimuli consisted of blocks of moving and static white point clouds (motion and static condition). In the motion condition, dots moved outward (outward motion) or inward (inward motion). (*A*–*C*) During all experiments, participants were instructed to maintain a central fixation point that was presented during all blocks. In the M/P experiment (*B*), participants additionally performed a decoy task, in which they detected contrast decrements that were randomly presented within the stimuli (example gray dot in stimulus on the left of the panel; *SI Appendix*).

A standard checkerboard experiment (Fig. 1*A*) showed comparable overall LGN responses to hemifield visual stimulations in the autism and control group (*SI Appendix*).

### Reduced mLGN Responses in the Autism Group as Compared to the Control Group for Stimuli Optimized for the mLGN.

**Fig. 2.**
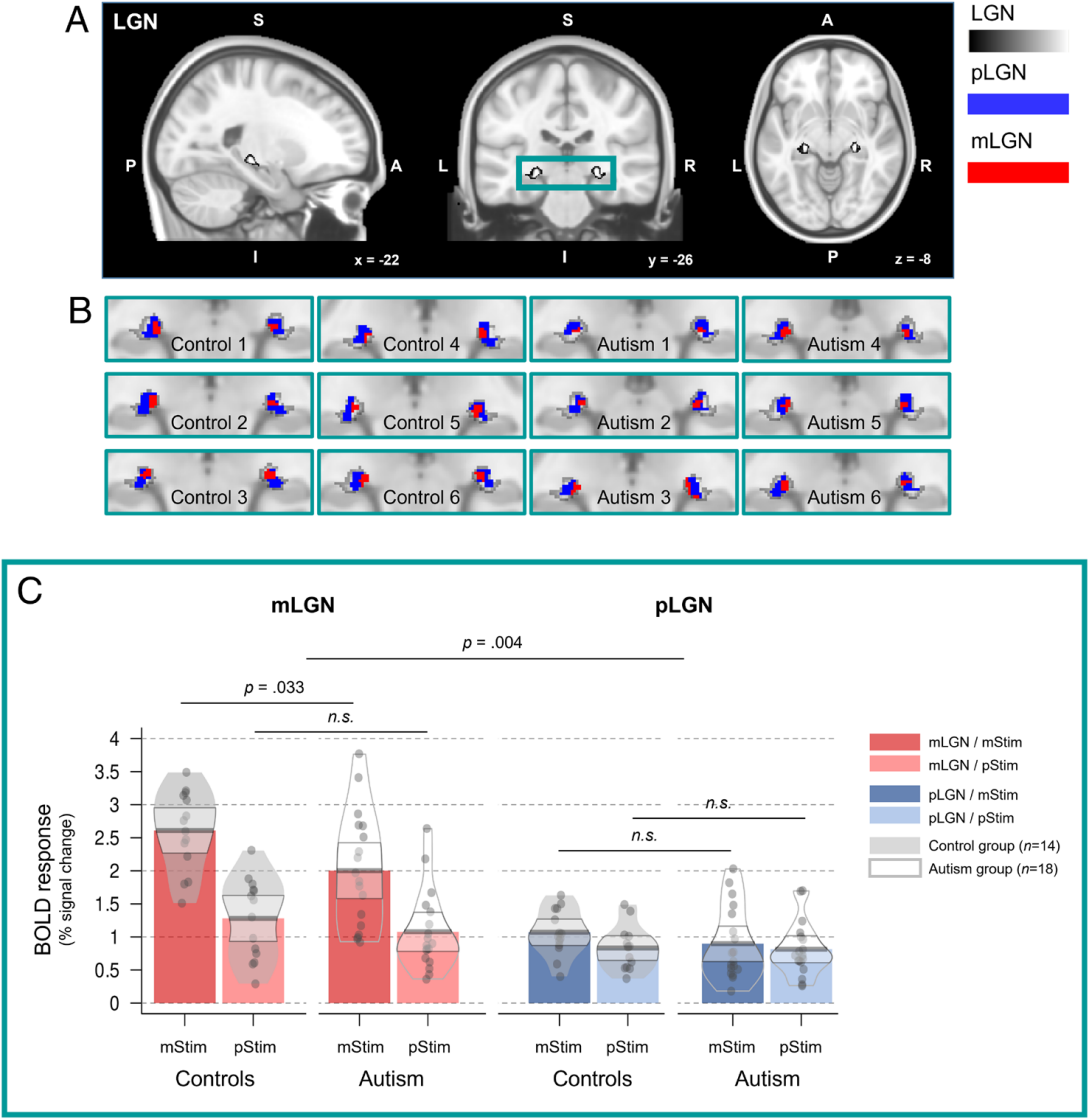
(*A*) To locate the individual LGNs, we used a high-resolution probabilistic mask of an independent LGN atlas (*SI Appendix*). (*B*) M/P-maps in example individuals of the control (Control 1 to 6) and the autism group (Autism 1 to 6) based on the M/P experiment. M/P-maps were defined by 20/80% volume thresholding (*SI Appendix*) within the individual LGN maps (gray). For these maps, mLGN voxels show a higher response preference for the mStim condition (red), whereas pLGN voxels show a higher preference for the pStim condition (blue). All maps are plotted on a standard Montreal Neurological Institute (MNI) brain. (*C*) Reduced mLGN BOLD responses in the autism group for mLGN optimized stimuli (mStim) as compared to controls (https://osf.io/w7pr4/ for individual results). Bars represent the bilateral mean BOLD response (% signal change). Beans represent the smoothed density curve showing the full data distribution. Within beans, dots represent the mean BOLD response of each participant. Bands represent the 95% CI around the mean. LGN = lateral geniculate nucleus; m = magnocellular; p = parvocellular; P = posterior; S = superior; A = anterior; I = inferior; L = left; R = right; x, y, z = coordinates in MNI space; Stim = Stimulus condition; n.s. = not significant.

To test our main hypothesis, we used the M/P experiment (Fig. 1*B*) with stimuli optimized to selectively activate the mLGN and pLGN (mStim and pStim conditions). An ANOVA with the between-subjects factor group (controls; autism) and the within-subject factors LGN subdivision (mLGN; pLGN), hemisphere (left; right), and stimulation type (mStim; pStim) showed a significant 3-way-interaction between the factors group x LGN subdivision x stimulation type [*F*(1,30) = 9.602, *p* = 0.004, *η^2^_p_*= 0.242] (Fig. 2 *A*–*C*). As expected, post-hoc independent *t*-tests revealed that the interaction was driven by significantly lower responses in the mLGN to the mStim condition in the autism as compared to the control group [*t*(30) = 2.235, *p* = 0.033, *g* = 0.83, two-tailed] (Fig. 2*C*). There were no significant group differences for the pLGN or the pStim condition (all *p* >/= 0.310; *g* </= 0.35, *SI Appendix*). The results confirm the hypothesis of a specific alteration of mLGN function in the autism group. The results were based on 18 autism and 14 control group participants. Single participant results are provided on osf (osf.io/w7pr4). Four additional controls and one autism group participant did not pass quality control for LGN subdivisions (*SI Appendix*). The results remained qualitatively the same using a linear mixed-effects model for analysis (*SI Appendix*).

### mLGN Responses to Coherent Motion in mLGN in Autism and Controls.

Next, we tested whether mLGN alterations in autism can also be revealed with a second experiment involving coherent motion. Coherent motion perception is thought to rely on the cerebral cortex and is performed not exclusively by the magnocellular visual system (7). Nevertheless, a recent study revealed higher responses in mLGN than pLGN to coherent motion using the same paradigm as we used here (Fig. 1*C*) (8). An ANOVA for the contrast motion > static for the between-subject factor group (controls; autism) and LGN subdivision (mLGN; pLGN) and hemisphere (left; right) as within-subject factors revealed no significant main effects or interactions (all *p*s >/= 0.06; *SI Appendix*). The lack of statistically significant enhanced mLGN responses to motion relative to pLGN was unexpected and the interaction between group and LGN subdivision was close to the significance threshold with a medium effect size [*F*(1,30) = 3.801, *p* = 0.061, *η^2^_p_* = 0.112]. We therefore performed exploratory post-hoc paired *t*-tests, which indicated as expected a higher response for the mLGN as compared to the pLGN for the contrast motion > static [*t*(13) = 3.440, *p* = 0.004, *d* = −0.79] in the control group. Conversely, for the autism group, this test was not significant (*p* = 0.960).

### Control Analyses.

The structural mLGN and pLGN volumes were comparable between the groups and there were no significant differences in head motion between the two groups in none of the experiments (*SI Appendix*).

## Discussion

The key finding of altered mLGN responses in autism for stimuli optimized to elicit mLGN responses is an empirical proof for the long-standing hypothesis of alterations of the magnocellular visual system in autism (4, 5). Many socio-communicative tasks such as recognising emotion or speech from a face involve the analysis of features associated with mLGN function (9). For example, communicative face movements correspond to rapid visual changes at large spatial scales (10, 11). This also includes face emotion and speech recognition, both of which are altered in autism (9, 11, 12, 13). In line with recent views (9), we speculate that differences in mLGN function might at least partly explain differences in social-communicative tasks. The potential influence of other cognitive mechanisms, like attention, should however also be considered (14). The present study could be a starting point for investigating in how far mLGN alterations contribute to autism social-cognitive symptoms (9).

Besides magnocellular layers, the mLGN includes koniocellular layers which are involved in motion processing via the dorsal stream (6, 7, 15). To date, it is impossible to dissociate them in humans in vivo. Since magnocellular cells dominate the mLGN (6), it is likely that the group differences found here are based on magno- rather than koniocells.

The sample size of this study is a result of the organizational, monetary, and technical challenges in conducting 7T-MRI studies with clinical populations. We anticipate that the new discoveries will generate larger studies across multiple labs to investigate the functional relevance of mLGN alterations for autism symptoms.

Our findings provide evidence for the still largely unexplored view of a critical role of altered sensory processing for autism (3, 9). Further, a transdiagnostic approach with clinical conditions that potentially overlap in symptoms and subcortical alterations [e.g., schizophrenia and dyslexia (8, 16)] could be a promising approach to investigate the relation of sensory processing alterations and social symptoms.

## Materials and Methods

Control and autism group participants were matched pairwise on age (± 4 y), sex, handedness, and full-scale IQ (IQ points ± 15 and > 85) (*SI Appendix*). All autism group participants had a formal diagnosis of an autism spectrum disorder (ASD; *SI Appendix*). The study was approved by the Ethics Committee of the Medical Faculty at the University Leipzig, Germany (file number 237/17-ek). All participants gave written informed consent in accordance with the Declaration of Helsinki and procedures approved by the Research Ethics Committee of the University of Leipzig. MR-images were acquired on a 7-Tesla Magnetom MRI system (Siemens Healthineers, Germany) with a 32-channel head coil. For details, Fig. 1 and *SI Appendix*, *Extended Methods*.

### Data Sharing.

The scripts used to generate stimuli for all experiments are publicly available [checkerboard, M/P experiment *SI Appendix*; coherent motion experiment see (8) and *SI Appendix*]. Scripts for fMRI analyses and individual LGN, mLGN, and pLGN maps are available in osf (osf.io/w7pr4). Raw MRI data cannot be made publicly available since sharing these data is not covered by the ethics clearance for all participants. Data of those participants who agreed to share their data can be made available upon contacting the first author.

## Supplementary Material

Appendix 01 (PDF)

## Data Availability

Anonymized Scripts for fMRI analyses and individual LGN, mLGN and pLGN maps data have been deposited in OSF https://osf.io/w7pr4/ (17)). Some study data available (Raw MRI data cannot be made publicly available since sharing these data is not covered by the ethics clearance for all participants. Data of those participants who agreed to share their data can be made available upon contacting the first author.). Previously published data were used for this work [The scripts used to generate stimuli for all experiments are publicly available (checkerboard, M/P experiment *SI Appendix*; coherent motion experiment (8); *SI Appendix*).].
