# Supplementary material for: Functional alterations of the magnocellular subdivision of the visual sensory thalamus in autism
Source: Proc Natl Acad Sci U S A. 2024 Nov 11;121(47):e2413409121. doi: 10.1073/pnas.2413409121 (PMC11588090; doi:10.1073/pnas.2413409121)
Supplement: Supplementary file 1 — Appendix 01 (PDF) [file pnas.2413409121.sapp.pdf]

PNAS

Supporting information for

Functional alterations of the magnocellular subdivision of the visual sensory thalamus in autism

Stefanie Schelinski* [a, b], Louise Kauffmann [b, c], Alejandro Tabas [b, d, e], Christa Müller-Axt [a, b], Katharina von Kriegstein [a, b]

Author affiliations:
[a] Chair of Cognitive and Clinical Neuroscience, Faculty of Psychology, Dresden University of Technology, Dresden 01187, Germany
[b] Max Planck Institute for Human Cognitive and Brain Sciences, Leipzig 04303, Germany
[c] Laboratoire de Psychologie et Neurocognition, Université Grenoble Alpes, Grenoble 38000, France
[d] Basque Center on Cognition, Brain and Language, San Sebastian 20009, Spain
[e] Ikerbasque, Basque Foundation for Science, Bilbao 48009, Spain

* Corresponding author: Stefanie Schelinski
Email: stefanie.schelinski@tu-dresden.de

This PDF file includes:

Supporting text
SI References

Supporting information text

Extended methods

Participants

The following descriptive data for the participants of the original sample refer to 19 control group and 19 autism group participants. In the original sample, each participant in the control group was pair-wise matched to the profile of one autism group participant with respect to chronological age (see below), gender (12 male, 7 female), handedness (17 right, 2 left-handed), and intelligence quotient (IQ, see below). The descriptive data for the participants of the final sample refer to 14 control group (8 male, 6 female) and 18 autism group participants (12 male, 6 female). For each characteristic, we report mean values (*M*) over group and standard deviation (*SD*) and results from independent *t*-tests along with effect sizes (Cohen's *d*).

Age
In the original and the final sample, the age ranged between 20 and 41 years of life in the control group and between 19 and 41 years of life in the autism group. Age was comparable between the groups (*Original sample*. Control group: *M* = 26.95, *SD* = 5.46; Autism group: *M* = 27.05, *SD* = 6.05; Group comparison: *t*(36) = -0.056, *p* = 0.955, *d* = 0.017. *Final sample*. Control group: *M* = 25.93, *SD* = 5.88; Autism group: *M* = 27.50, *SD* = 5.89; Group comparison: *t*(30) = -0.749, *p* = 0.460, *d* = 0.267).

Assessment of cognitive skills
IQ was assessed using the German adapted version of the Wechsler Adult Intelligence Scale (WAIS-IV [1] or WAIS-III [2] if data were available from previous study participation). All participants had an IQ within the normal range or above (IQ > 85) and IQ was comparable between the groups (*Full-scale IQ, original sample*. Control group: *M* = 111.58, *SD* = 10.69; Autism group: *M* = 110.42, *SD* = 12.20; Group comparison: *t*(36) = 0.311, *p* = 0.758, *d* = -0.101. *Full-scale IQ, final sample*. Control group: *M* = 113.00, *SD* = 11.13; Autism group: *M* = 111.22, *SD* = 12.03; Group comparison: *t*(30) = 0.428, *p* = 0.671, *d* = -0.154). Additionally, groups showed comparable concentration performances (d2 test of attention [3]). (*Concentration performance [KL], original sample*. Control group: *M* = 106.89, *SD* = 7.94; Autism group: *M* = 107.74, *SD* = 9.50; Group comparison: *t*(36) = -0.297, *p* = 0.769, *d* = 0.097. *KL, final sample*. Control group: *M* = 105.50, *SD* = 6.77; Autism group: *M* = 108.33, *SD* = 9.40; Group comparison: *t*(30) = -0.951, *p* = 0.349, *d* = 0.345).

Assessment of reading and writing skills
Alterations in LGN functioning have been associated with developmental dyslexia [4], a condition associated with low reading skills that often goes along with poor spelling skills [5]. None of the participants reported to have ever received a dyslexia diagnosis or poor reading or spelling skills. To control for potential effects of unrecognised dyslexia cases in our samples, we additionally assessed reading and spelling skills using standard tests for dyslexia assessment in Germany (Reading: German reading speed and comprehension test (LGVT 6-12 [6]) and rapid automized naming (RAN [7]); Spelling: Spelling test (RT [8])). There were no group differences in reading and spelling skills between the autism and the control group. All participants performed at least within the normal range (T >/= 40) in the LGVT-subscores reading speed and reading comprehension, indicating that all of the participants had average reading skills (*Reading speed (t), original sample*. Control group: *M* = 56.21, *SD* = 10.02; Autism group: *M* = 56.05, *SD* = 10.57; Group comparison: *t*(36) = 0.047, *p* = 0.963, *d* = -0.018. *Reading speed (t), final sample*. Control group: *M* = 58.07, *SD* = 11.03; Autism group: *M* = 56.72, *SD* = 10.46; Group comparison: *t*(30) = 0.353, *p* = 0.726, *d* = -0.126. *Reading comprehension (t), original sample*. Control group: *M* = 60.16, *SD* = 9.14; Autism group: *M* = 63.05, *SD* = 10.92; Group comparison: *t*(36) = -0.886, *p* = 0.381, *d* = 0.287. *Reading comprehension (t), final sample*. Control group: *M* = 62.36, *SD* = 9.56; Autism group: *M* = 63.83, *SD* = 10.68; Group comparison: *t*(30) = -0.406, *p* = 0.688, *d* = 0.145). Additionally, none of the participants performed 2SD below the overall group's mean in the RAN and spelling tests (*RAN composite score letters and numbers [RANln] (time sec), original sample*. Control group:

$M = 18.59$, $SD = 2.44$; Autism group: $M = 18.27$, $SD = 3.56$; Group comparison: $t(36) = 0.322$, $p = 0.749$, $d = -0.095$. *RANln (time sec), final sample*. Control group: $M = 18.58$, $SD = 2.38$; Autism group: $M = 17.98$, $SD = 3.42$; Group comparison: $t(30) = 0.558$, $p = 0.581$, $d = -0.204$. *Spelling test, original sample*. Control group: $M = 100.26$, $SD = 13.03$; Autism group: $M = 108.21$, $SD = 12.61$; Group comparison: $t(30) = -1.910$, $p = 0.064$, $d = 0.620$. *Spelling test, final sample*. Control group: $M = 103.86$, $SD = 10.93$; Autism group: $M = 108.00$, $SD = 12.94$; Group comparison: $t(36) = -0.960$, $p = 0.345$, $d = 0.346$).

Vision and hearing

All participants reported normal or corrected-to-normal visual acuity and no disorders associated with vision. Visual acuity was confirmed with the Freiburg Visual Acuity Test (FrACT3) [9, 10] with a cut-off of +0.1 binocular logMAR. All participants reported normal hearing abilities and no limitations or disorders associated with the ear or hearing. Normal hearing abilities were confirmed with pure tone audiometry (hearing level equal or above 25 dB at the frequencies of 250, 500, 1000, 1500, 2000, 3000, 4000, 6000, and 8000 Hz tested in each ear separately).

Autism diagnostics

Participants in the autism group had previously received a formal clinical diagnosis of Asperger's syndrome (12 male, 7 female) according to the diagnostic criteria of the International and Statistical Classification of Diseases and Related Health Problems (ICD-10) [5]. Additionally, the diagnoses for all participants in the autism group (except for 1 participant) were corroborated with the Autism Diagnostic Observation Schedule (ADOS [11]; German version by [12]) and, if caregivers were available ($n = 10$), additionally with the Autism Diagnostic Interview-Revised (ADI-R [13]; German version by [14]) and the Social Communication Questionnaire (SCQ [15]; German version by [16]). Mean scores (cut-offs for autism / autism spectrum): *ADOS.* Social interaction and communication: $M = 10.33$ (12 / 7), $SD = 2.99$; Social interaction: $M = 6.94$ (7 / 4), $SD = 2.26$; Communication: $M = 3.39$ (3 / 2), SD = 1.19. *ADI-R*. Social interaction and communication: $M = 27.10$, $SD = 11.22$; Social interaction: $M = 17.70$ (17), $SD = 7.49$; Communication: $M = 9.40$ (8), $SD = 4.06$. *SCQ*. Total: $M = 19.56$ (15), $SD = 8.28$.

Recruitment

We recruited autistic participants via autism outpatient clinics and announcements in communities for people on the autism spectrum, such as self-help groups and online fora. We only included participants into the autism group who could provide a clinical diagnosis. That means that independent clinical experts made the diagnoses of all autistic participants before participating in the study. We recruited the control group participants from the participant database of the Max Planck Institute for Human Cognitive and Brain Sciences Leipzig. The database contains participants who have contacted the institute because they are interested in taking part in scientific studies or have been recruited in the context of other studies. The database contains volunteers with e.g., different age ranges and different socioeconomic status or educational backgrounds.

Further criteria

All participants were free of psychotropic medication. Participants in the control group reported to have no neurological or psychiatric history and no family history of autism. None of the control group participants exhibited a clinically relevant number of traits associated with autism as assessed by the autism spectrum quotient (AQ [17]; German version adapted from [18]), i.e., all total scores </= 23. A total score of 32+ is considered a useful cut-off for distinguishing individuals who have clinically relevant levels of traits associated with autism spectrum [17] (*Total score, original sample*. Control group: $M = 13.68$, $SD = 4.90$; Autism group: $M = 35.16$, $SD = 10.22$; Group comparison: $t(36) = -8.258$, $p < 0.001$, $d = 2.680$. *Total score, final sample*. Control group: $M = 13.79$, $SD = 4.70$; Autism group: $M = 35.56$, $SD = 10.37$; Group comparison: $t(30) = -7.280$, $p < 0.001$, $d = 2.704$). Additionally, all participants met all local safety requirements for participating in an ultra-high field 7T-MRI study (e.g., no metal implants, free of tattoos, no dental amalgam restorations). Handedness was assessed using the Edinburgh inventory [19]. All participants were native German speakers.

All participants received reimbursement for their participation. Since there were no a-priori data for a formal sample size estimation, we attempted to maximise the number of clinical participants we could recruit in a given time (approximately one year).

Experiments

Experimental procedure

All MRI experiments were performed within one session on the same day. The control and autism group participants underwent all experiments under the same testing conditions and in the same following experimental order: 1. Diagnostics (including psychological tests, hearing measurements, etc.), 2. MRI, 2.1. Checkerboard experiment, 2.2. M/P experiment, 2.3 Coherent motion experiment. If possible, diagnostics and fMRI were performed on separate days. The fMRI experiments were part of a study in which participants additionally performed computer tests and a diffusion-weighted (DWI) MRI scan. Results from these study parts will be reported elsewhere. The experiments took place at the Max Planck Institute for Human Cognitive and Brain Sciences (Leipzig, Germany). For participants who never had an MRI before, we conducted a mock MRI to familiarise them with the MRI environment. During MRI, participants wore hearing protection plugs and received a foam padding around the head to reduce head motion.

Stimulus presentation

Stimuli were presented to the participants via front-projection onto a translucent screen positioned above the participant's chest. Participants saw the screen via a mirror that was placed on the MRI coil over the subjects' eyes (total viewing distance approximately 35 cm and 18 x 16 degrees of visual angle). Before the actual MRI experiments the positioning of the mirror was optimised and we made sure that the whole visual field was covered using an example picture from the checkerboard experiment. Stimuli were generated on the fly using Psychtoolbox [20, 21] implemented in GNU Octave (version 4.2.0 [22]). Stimuli were presented with a refresh rate of 60 Hz.

Functional MRI experiments

Checkerboard experiment

Stimuli. In the checkerboard experiment, we presented alternating hemifield stimuli (Fig. 1A). Stimuli consisted of checkerboards that covered half of the screen, while the other half of the screen contained background grey luminance (50% contrast, luminance 1019 cd/m^2). Checkerboards had a radial check pattern with a check size of 15° polar angle. Eccentricity of the checks was scaled according to the formula $s = 0.05 \times r^{0.8}$ with s is the check size and r is the distance from fixation in degrees of visual angle. The fixation point participants were asked to attend was a white dot with a 0.2° angle and appeared at the centre of the screen during the whole runs. For more details on the stimuli see [23].

Experimental design. One block lasted 16 sec containing either the left or the right half checkerboard presentation (16 blocks in total, 8 per side). The total duration of the experiment was approximately 5 min.

M/P experiment

Stimuli. M/P-stimuli covered the whole screen (Fig. 1B). To avoid sharp visual edges at the stimulus boundaries, the outer borders of the stimuli faded into grey. For mStim and pStim condition stimuli, sinusoidal gratings were presented at one of 6 different orientations (0°, 30°, 60°, 90°, 120°, or 150°). In order to drive populations of LGN neurons with different spatial receptive fields, the orientation changed to a new orientation every 3 sec within a block. The blank stimulus was a grey screen of mean luminance. The fixation point participants were asked to attend during the whole experiment was a white dot with a 0.2° angle and appeared at the centre of the screen during the whole runs. For more details on the stimuli see [23].

Experimental design. Blocks were presented in pseudorandom order within 3 runs. One run consisted of 15 blocks (6 mStim, 6 pStim, 3 blank) with the constrain that two mStim or two pStim

blocks were never presented adjacently. Each block lasted 16 sec. The total duration of the experiment was approximately 15 min.

Target detection task. In addition to maintaining a central fixation point during all blocks, participants performed a target detection task during the mStim and pStim blocks. They were asked to count the number of circular targets that appeared within each block (0-3). Participants had to decide after each block (response window of 1.5 sec) via a button box, whether 0, 1, 2 or 3 targets appeared within the block. Targets were 300 ms long 2-dimensional Gaussian contrast decrements. Within each block, targets appeared at random times at any location within the stimulus. To familiarise the participants with the stimulus material and the target detection task, they received a training consisting of 2 mStim and 2 pStim blocks containing targets as well as a blank block before the actual experiment started.

Coherent motion experiment

Stimuli. In both conditions, 250 white dots with a radius of 0.1° were presented against a black background (Fig. 1C). In the motion condition, the dots moved radially at a speed of 4.7 deg/sec with 100% coherence within a circular aperture of 17°. Radial motion was chosen to facilitate central fixation and to stimulate a broad spectrum of motion direction selective cells [24]. The fixation point participants were asked to attend was a grey dot that appeared at the centre of the screen during all runs. For more details see [4, 25].

Experimental design. Dot clouds either moved inwards or outwards (each for half of the motion blocks). In the static condition, dots were displayed at random locations and remained stationary. One block lasted 16 sec (16 blocks in total, 8 blocks per condition). The total duration of the experiment was approximately 5 min.

Image acquisition

Functional MRI

In all three fMRI experiments, we used echo planar imaging (EPI) with a high resolution of 1.25 mm x 1.25 mm x 1.2 mm (no gap); 40 transverse slices; partial brain coverage covering the LGN and visual cortex; TE = 16 ms; TR = 2000 ms). EPI sequences had the following further imaging parameters: flip angle = 80°, FoV = 152 x 170 x 69 mm^3, echo spacing = 0.78 ms, acquisition bandwidth = 1476 Hz/Px, GRAPPA = 3, Partial Fourier = 6/8 in phase-encoding direction. For one control group participant the flip angle was adjusted to 72° due to restrictions in energy absorption (i.e., specific absorption rate) typically associated with high-field MRI. To correct images for geometric distortions induced by magnetic field inhomogeneity, we acquired in each MRI session two gradient-echo datasets (Δ_{TE} = 1.02 ms) from which session-specific B0 field-maps (voxel displacement) were computed. All images were acquired continuously (checkerboard experiment: 136 volumes; M/P experiment: 3 runs of 144 volumes; coherent motion experiment: 130 volumes). To optimise fMRI data quality, we recorded cardio-respiratory data during all experiments.

Cardio-respiratory data acquisition during functional MRI

Including cardio-respiratory information in the fMRI data analyses enhances the signal-to-noise ratio in the LGN by accounting for physiological noise in the BOLD signal during data processing [26]. We recorded cardio-respiratory data during all three fMRI experiments using an MRI-compatible Biopac System (Biopac Systems, Inc., CA, USA). We acquired cardio data via a pulse oximeter that was placed on the participants' non-dominant index finger. We recorded respiratory data through thoracic movements using a non-electrical pressure pad. The pad was placed on the participants' chest in combination with a respiration transducer. We sampled cardio-respiratory data at a sampling rate of 100 Hz.

Structural MRI

For anatomical images, we used a standard T1-weighted 3D magnetization-prepared rapid gradient echo sequence (M2PRAGE) (whole brain coverage, 0.7 mm isotropic resolution) [27] with the following imaging parameters: TE = 2.45 ms, TR = 5000 ms, TI1/TI2 = 900/2750 ms, α1/α2 = 5/3°, FoV = 224 x 224 x 168 mm3, echo spacing = 6.8 ms, readout bandwidth = 250 Hz/Px,

GRAPPA = 2, Partial Fourier = 6/8 in phase-encoding direction. MRI-acquisition time for the structural images was 10 min 57 sec.

Data analyses

The pipeline for data analyses is based on procedures described in previous studies [4, 23]. The data analyses pipeline contained the following steps: (1) Data pre-processing, (2) LGN segmentation, (3) mLGN and pLGN definition, (4) Quality control, (5) Signal change extraction and statistical analyses.

Software

For pre-processing of the MRI data and first level statistical analyses we used standard procedures implemented in SPM (version 12, Wellcome Centre for Human Neuroimaging, London, UK) in a Matlab environment (version 9.3, The MathWorks Inc., Natick, USA). Pipelines for anatomical LGN segmentation were coded with nipype (version 1.8.5 [28]) and NiBabel (version 4.0.1 [29] and performed using SPM (as above), FSL (version 6.03, FMRIB Centre, Oxford, UK) and ANTs (version 2.3.5 [30]). We also used NiBabel for quality control of the identified M/P subdivisions. For percent signal change extraction, we used an in-house toolbox. For further analyses of the fMRI data and for analysing behavioural data we used SPSS software (version 24, IBM SPSS Statistics, New York, USA). Physiological parameters were obtained using the PhysIO toolbox [31]. For visualisation we used R [32] and FSLeyes (version 0.31.2, FMRIB Centre, Oxford, UK, implemented in FSL version 6.0.3).

Pre-processing

We realigned all individual images of the M/P experiment and the coherent motion experiment to the first volume of the checkerboard experiment. To correct for motion artefacts and EPI distortions, images were unwarped based on the fieldmaps. We also co-registered the whole-brain EPI image to the first volume of the checkerboard experiment. This co-registered image served as reference image for registering the structural to the functional data. Next, the realigned and unwarped functional data in native space were spatially smoothed with a Gaussian filter with a full width half maximum (FWHM) matching the voxel size (i.e., 1.25 x 1.25 x 1.2mm). Time series of each voxel were high-pass filtered (1/128 Hz cut-off) to remove low-frequency noise and signal drift. For quality control (see below), we also normalised the realigned and unwarped functional data into MNI-space (Montreal Neurological Institute) using non-linear registration to an 1mm MNI brain template. These registered data were also smoothed with a Gaussian filter with FWHM matching the voxel size (i.e., 1.25 x 1.25 x 1.2 mm) and high-pass filtered at 1/128 Hz cut-off.

LGN definition

LGN segmentation

We segmented the left and right LGN in each participant using an independent publicly available high-resolution 7T probabilistic atlas [33, 34]. LGN-maps from the probabilistic atlas were registered to the individual T1 native images. The individual LGN masks were then registered to the functional imaging data. For segmenting the left and right LGN, we first extracted the brain, i.e., removed the skull and non-brain areas from the native T1 images from each participant ("skull stripping") using the FSL-tool BET. Due to the noisy contrast for voxels corresponding to air of the MP2RAGE T1 images, FSL BET was unable to fully remove all the voxels corresponding to the skull in all subjects. To remove the remaining non-brain areas from the images we used SPM to segment the CSF, white and grey matter, added the masks, and binarised the combined brain mask with fslmaths. We further skull stripped the images applying the brain mask with fslmaths. In the next step, we normalised the skull stripped T1 images to MNI space using a 0.4mm isotropic template [33, 34]. We used ANTs to compute the transformations between the skull stripped T1 images (fixed image) with the MNI template (moving image), and between individual T1 spaces and MNI space. We then used the transforms between the MNI and T1 spaces to map right and left LGN provided in a probabilistic atlas of the human to the space of the participants [34] (at a threshold of 35% overlap). Individual right and left LGN masks were then registered to the functional image data. The transformations were computed using a standard three-step procedure (rigid followed by affine followed by non-linear symmetric normalisation).

mLGN and mLGN definition

We defined individual left and right mLGN and pLGN subdivisions following a common procedure using the M/P experiment [4, 23]. We computed individual Beta M/P-maps using a general linear model (GLM). To compute Beta M/P-maps in native space, we subtracted the Beta maps obtained from the GLM estimation corresponding to the mStim and pStim condition in the M/P experiment. This procedure follows that those voxels with larger values on the Beta M/P-maps correspond to a higher response preference for the mStim condition, while voxels with lower values correspond to a higher response preference for the pStim condition [23]. To confine these maps to relevant voxels within the LGN, individual Beta M/P-maps were then masked with the previously defined individual right and left LGN masks (see LGN segmentation above). The mLGN was defined as the 20% of voxels with the largest Beta-values for the difference between mStim – pStim condition. The pLGN was defined as the remaining 80% of voxels. The 20/80% volume allocation criterion is based on previous histological studies which show that the proportion of mLGN and pLGN- neurons in the human brain fall within these bounds [35].

Quality control of mLGN and pLGN subdivisions

To verify that we accurately identified mLGN and pLGN, we checked: (1) The volume of each subdivision in each participant, and (2) that the centre of mass (CoM) of the mLGN was more medial than the CoM of the pLGN. In total, we excluded 5 control group and 1 autism group participants, because they did not meet at least one of the quality control criteria.

Volume of mLGN and pLGN

To make sure that the LGN subdivisions were of plausible size, we computed the volume of the mLGN and pLGN subdivisions in each participant for each hemisphere. We excluded 4 participants (3 controls and 1 autism) whose mLGN or pLGN was 2 SD smaller than for the overall group mean (see control analyses below for LGN-sizes for the final samples).

Centre of mass (CoM)

We checked, for each participant, whether the CoM of the mLGN was more medial than the CoM of the pLGN (in native space). This criterion is based on prior anatomical knowledge [23, 34]. To compute the CoMs, we first masked the M/P contrast obtained with the functional localiser with our estimation of the m- and p-masks. We then computed the CoM of each subdivision. The CoMs were normalised as CoM_norm_M/P = (CoM_M/P - CoM_LGN) / extent_LGN. We excluded 3 control group and 1 autism group participants for whom the mLGN was more lateral than the pLGN. One of those autism group and one of those control group participants additionally did not meet inclusion criteria for the LGN extent (see above).

Signal change extraction

We extracted individual Beta estimates from all voxels for all experimental conditions from the left and right LGN (i.e., checkerboard experiment) and the left and right mLGN and pLGN maps (i.e., M/P experiment and coherent motion experiment). Beta estimates were converted to % signal change (PSC) that we used as dependent variable in all statistical analyses. The PSC was computed as follows: PSC = $ß_{condition}$ x SF / $ß_{constant}$ x 100 (with $ß_{condition}$ = parameter estimate of the condition of interest, $ß_{constant}$ = parameter estimate for the constant term, SF = scale factor of the matrix) [36].

fMRI data analyses

For statistical analyses, we used the mean PSC for a given region (in native space) and condition. For all analyses, statistical parametric maps were generated by modelling the evoked haemodynamic response for the different conditions as boxcar functions convolved with a canonical haemodynamic response function using the general linear model (GLM) [37]. To account for motion and variance due to cardio-respiratory differences, we included 6 motion parameters derived from data realignment (i.e., three translation and three rotation) and 16 physiological parameters derived from the PhysIO toolbox [31] as regressors of no interest in the first-level analyses. Physiological regressors included models of heart rate variability (1 regressor [38]), respiratory volume per time (1 regressor [39]) and cardiac (6 regressors) and respiratory phases (8 regressors) computed using

the Fourier-expansions of different order, based on RETROICOR [40]. Including motion and models based on cardio-respiratory information as regressors for 7T fMRI substantially increases the signal-to-noise ratio in the LGN by increasing BOLD sensitivity [26].

Statistical thresholds

We considered effects as significant at $p < 0.05$. For main analyses, we used a mixed-design measures analyses of variance (ANOVAs) for the percent signal change (PSC) as dependent variable for the between-subject factor group (control group; autism group) and the within-subject factors hemisphere (left; right) and stimulation side (left hemifield; right hemifield) in the checkerboard experiment, LGN subdivision (mLGN; pLGN), hemisphere (left; right) and stimulation type (mStim; pStim) in the M/P experiment. For the coherent motion experiment, we used the PSC for the condition contrast motion > static as dependent variable and LGN subdivision (mLGN; pLGN) and hemisphere (left; right) as within-subject factors. We used independent *t*-tests for (post-hoc) between-group and dependent *t*-tests for within-group comparisons. All statistical tests are reported two-tailed if not stated otherwise. We report partial eta square (η^2_p) and Cohen's *d* or Hedges' *g* (for samples of different size) as effect sizes along the results. All fMRI analyses contain data from the final sample, i.e., 14 control and 18 autism group participants.

Detailed results

Checkerboard experiment

An ANOVA for the between-subjects factor group (controls; autism) and the within-subject factors hemisphere (left; right) and stimulation site (right hemifield; left hemifield) revealed no significant main effect of group ($F(1,30) = 2.766$, $p = 0.107$, $\eta^2_p = 0.084$) and no interactions with the factor group (all *p*'s > 0.148). This indicates that overall LGN responses to visual stimulation were similar between the groups.

M/P experiment

ANOVA. Further, there were 2-way-interactions for group x LGN subdivision ($F(1,30) = 5.319$, $p = 0.028$, $\eta^2_p = 0.151$), group x stimulation type ($F(1,30) = 8.656$, $p = 0.006$, $\eta^2_p = 0.224$) and LGN subdivision x stimulation type ($F(1,30) = 584.245$, $p < 0.001$, $\eta^2_p = 0.951$) and main effects for LGN subdivision ($F(1,30) = 155.636$, $p < 0.001$, $\eta^2_p = 0.838$) and stimulation type ($F(1,30) = 182.112$, $p < 0.001$, $\eta^2_p = 0.859$). There was no main effect of group ($F(1,30) = 1.816$, $p = 0.188$, $\eta^2_p = 0.057$) and no further significant main effects or interactions.

Linear mixed-effects model. Data analyses. We used a linear mixed-effect model as an alternative approach to back up our main study findings in the M/P experiment. These models preserve the statistical power granted by having repeated measurements while accounting for variabilities of the main effects across participants. We defined the three main types of variables specified in a linear mixed-effects model (i.e., response, explanatory and grouping variables) as follows: In all models, we used the PSC as response variable. In each model we considered one or more of the following explanatory variables: 1) LGN subdivision (mLGN / pLGN), 2) stimulation type (mStim / pStim), and 3) group (control or autism). The grouping variables were always the participant's ID, so that repeated measurements from a single participant are grouped together rather than taken as independent samples. We first examined effects within each group (control/autism) independently: 1) differences in PSC in the mLGN and pLGN during the given stimulation type and 2) differences in PSC elicited by each stimulation type in a given LGN subdivision. This yields a total of four statistical tests per group (8 within-group comparisons in total). After characterising the activation profiles in each group, we systematically tested for interactions between the two groups by training four additional mixed-effects models, corresponding to the four statistical tests performed per each group. The effect size and statistical significance are assessed using the statistics of the slope of the fixed-effects variable or interaction, respectively. All significant *p*-values reported in this analysis are Holm-Bonferroni corrected (i.e., 8 comparisons for within group and LGN subdivision / stimulation type comparisons and 4 comparisons for the interactions between the two groups). Results. The control and the autism group both showed enhanced PSC for the mStim as compared to the pStim condition within the mLGN (*p*s < 0.001, autism group: $b = 0.925$ [0.729, 1.120]; control group: $b = 1.330$ [1.160, 1.499]). This enhanced response within the mLGN was significantly greater for the control as compared to the autism group (i.e., there was a stimulation type x group

interaction for the mLGN: $p = 0.003$; $b = 0.404$ [0.147, 0.662]). Similarly, the mStim condition elicited enhanced responses in the mLGN as compared to the pLGN within both groups (ps < 0.001; autism group: $b = 1.104$ [0.871, 1.338]; control group: $b = 1.538$ [1.309, 1.767]). For this effect, there was a significant LGN subdivision x group interaction, indicating a stronger response in the control as compared to the autism group ($p = 0.010$; $b = 0.433$ [0.113, 0.754]). Within the control group, there was also enhanced PSC for the mStim as compared to the pStim condition within the pLGN ($p < 001$; $b = 0.239$ [0.0.148, 0.330]). There was no such significantly enhanced response for the pLGN within the autism group ($p = 0.153$, uncorrected; $b = 0.083$ [-0.341, 0.201]) and no stimulation x group interaction ($p = 0.042$; $b = 0.156$ [0.006, 0.306]). The pStim condition elicited enhanced responses in the mLGN as compared to the pLGN within the control group only ($p < 0.001$; $b = 0.448$ [0.246, 0.650]) and there was no significant LGN subdivision x group interaction for this effect ($p = 0.140$, uncorrected; $b = 0.185$ [-0.64, 0.434]).

Coherent motion experiment

Besides the main findings described in the main text, the effects for the ANOVA for the condition contrast motion > static for the between-group factor group (controls; autism) and LGN subdivision (mLGN; pLGN) and hemisphere (left; right) as within-subject factors were as follows (all non-significant): Main effect of group, $F(1,30) = 2.076$, $p = 0.065$, $\eta^2_p = 0.065$; Main effect LGN subdivision, $F(1,30) = 3.505$, $p = 0.071$, $\eta^2_p = 0.105$; Main effect hemisphere, $F(1,30) = 1.401$, $p = 0.246$, $\eta^2_p = 0.045$; Interaction group x LGN subdivision, $F(1,30) = 3.801$, $p = 0.061$, $\eta^2_p = 0.112$; Interaction group x hemisphere, $F(1,30) = 0.735$, $p = 0.398$, $\eta^2_p = 0.024$; Interaction LGN subdivision x hemisphere, $F(1,30) = 1.248$, $p = 0.266$, $\eta^2_p = 0.041$; Interaction group x LGN subdivision x hemisphere, $F(1,30) = 0.002$, $p = 0.961$, $\eta^2_p = 0$.

Control analyses

Size of LGN subdivisions

The average volume of the LGN for the control group (left LGN: 127.71 mm^3, right LGN: 135.93 mm^3) and the autism group (left LGN: 134.78 mm^3, right LGN: 140.72 mm^3) was comparable to the LGN sizes reported previously [34, 35]. Independent t-tests revealed that there were no significant group differences for the left and right LGN volumes between the final samples of the control ($n = 14$) and the autism group ($n = 18$) (left LGN: ($t(30) = -1.155$, $p = 0.257$, $g = 0.425$; right LGN: ($t(30) = -0.713$, $p = 0.482$, $g = 0.257$).

Head motion

To make sure that group differences are not due to group differences in head motion, we performed group comparisons for the motion parameters maximum translational displacement (TD), maximum rotational displacement (RD) and framewise displacement (FD, see [23] for more details on the calculation of these parameters). All motion parameters were derived from data realignment in SPM (see above). Independent t-tests for all motion parameters (i.e., TD, RD, and FD) from all experiments show that there were no significant group differences in head motion between the final samples of the control ($n = 14$) and the autism group ($n = 18$) (Checkerboard experiment: all p's > 0.07, all g's < 0.68; M/P experiment: all p's > 0.13, all g's < 0.54; Coherent motion experiment: all p's > 0.29, all g's < 0.37).

Response rate M/P experiment

Behavioural performance in the M/P experiment were available for 14 control group and 16 autism group participants. Response rates in the decoy task were comparable between groups ($t(28) = 0.833$, $p = 0.412$, $g = -0.305$). Mean response rates in the control group were 43,7% in the control group and 34,3% in the autism group. Potential reasons for such relatively low performance in the decoy task as compared to the original study [23] are discussed elsewhere [4].

SI References

1. F. Petermann, *Wechsler Adult Intelligence Scale – Fourth Edition (WAIS-IV). Deutsche Version* (Pearson Assessment, 2012).
2. M. von Aster, A. Neubauer, R. Horn, *Wechsler Intelligenztest für Erwachsene (WIE)* (Harcourt Test Services, 2006).
3. R. Brickenkamp, *Test d2 - Aufmerksamkeits-Belastung-Test (d2)* (Hogrefe, 2002).
4. C. Müller-Axt, L. Kauffmann, C. Eichner, K. von Kriegstein, Dysfunction of the magnocellular subdivision of the visual thalamus in developmental dyslexia. *Brain,* awae235, in press.
5. World Helath Organziation, *International Statistical Classification of Diseases and Related Health Problems (ICD-10)* (World Health Organization, 2004).
6. W. Schneider, M. Schlagmüller, M. Ennemoser, *Lesegeschwindigkeits- und -verständnistest für die Klassen 6 - 12: LGVT 6 – 12* (Hogrefe, 2007).
7. M. B. Denckla, R. G. Rudel, Rapid Automatized Naming (RAN) - Dyslexia differentiated from other learning-disabilities. *Neuropsychologia* **14**, 471-479 (1976).
8. M. Kersting, K. Althoff, *Rechtschreibungstest (RT)* (Hogrefe, 2004).
9. M. Bach, The Freiburg Visual Acuity Test - Automatic measurement of visual acuity. *Optom. Vis. Sci.* **73**, 49-53 (1996).
10. M. Bach, The Freiburg Visual Acuity Test - Variability unchanged by post-hoc re-analysis. *Graefes Arch. Clin. Exp. Opthalmol.* **245**, 965-971 (2007).
11. C. Lord et al., The autism diagnostic observation schedule-generic: A standard measure of social and communication deficits associated with the spectrum of autism. *J. Autism Dev. Disord*. **30**, 205-223 (2000).
12. D. Rühl, S. Bölte, S. Feineis-Matthews, F. Poustka, *Diagnostische Beobachtungsskala für Autistische Störungen (ADOS)* (Verlag Hans Huber, 2004).
13. C. Lord, M. Rutter, A. Le Couteur, Autism Diagnostic Interview-Revised: A revised version of a diagnostic interview for caregivers of individuals with possible pervasive developmental disorders. *J. Autism Dev. Disord*. **24**, 659-685 (1994).
14. S. Bölte, D. Rühl, G. Schmötzer, F. Poustka, *Diagnostisches Interview für Autismus – Revidiert (ADI-R)* (Verlag Hans Huber, 2003).
15. M. Rutter, A. Bailey, C. Lord, *Social Communication Questionnaire (SCQ)* (Western Psychological Services, 2003).
16. S. Bölte, F. Poustka, *Fragebogen zur Sozialen Kommunikation (FSK)* (Verlag Hans Huber, 2006).
17. S. Baron-Cohen, S. Wheelwright, R. Skinner, J. Martin, E. Clubley, The autism-spectrum quotient (AQ): Evidence from Asperger syndrome/high-functioning autism, males and females, scientists and mathematicians. *J. Autism Dev. Disord*. **31**, 5-17 (2001).
18. C. M. Freitag *et al.*, Evaluation der deutschen Version des Autismus-Spektrum-Quotienten (AQ) - die Kurzversion AQ-k. *Z. Klin. Psychol. Psychother.* **36**, 280-289 (2007).
19. R. C. Oldfield, The assessment and analysis of handedness: The Edinburgh inventory. *Neuropsychologia* **9**, 97-113 (1971).
20. D. G. Pelli, The VideoToolbox software for visual psychophysics: Transforming numbers into movies. *Spat. Vis.* **10**, 437-442 (1997).
21. D. H. Brainard, The psychophysics toolbox. *Spat. Vis.* **10**, 433-436 (1997).
22. J. Eaton, S. Bateman, S. Hauberg, H. Wehbring, *GNU Octave Version 4.2.0 Manual: A high-level interactive language for numerical computations* (Free Software Foundation, 2016).
23. R. N. Denison, A. T. Vu, E. Yacoub, D. A. Feinberg, M. A. Silver, Functional mapping of the magnocellular and parvocellular subdivisions of human LGN. *Neuroimage* **102**, 358-369 (2014).
24. K. M. O' Craven, B. R. Rosen, K. K. Kwong, A. Treisman, R. L. Savoy, Voluntary attention modulates fMRI activity in human MT-MST. *Neuron* **18**, 591-598 (1997).
25. C. Müller-Axt, L. Kauffmann, Data from "Data Repository: Dysfunction of the magnocellular subdivision of the visual thalamus in developmental dyslexia". Open Science Framework. Available at https://osf.io/bge75/. Deposited 29 May 2024.

26. C. Hutton, *et al.*, The impact of physiological noise correction on fMRI at 7 T. *Neuroimage* **57**, 101-112 (2011).
27. J. P. Marques *et al.*, MP2RAGE, a self bias-field corrected sequence for improved segmentation and T-mapping at high field. *Neuroimage* **49**, 1271-1281 (2010).
28. K. Gorgolewski *et al.*, Nipype: A flexible, lightweight and extensible neuroimaging data processing framework in python. *Front. Neuroinform.* **5**, 13 (2011).
29. M. Brett *et al.*, *nipy/nibabel (4.0.0)* (Zenodo, 2022).
30. B. B. Avants, N. J. Tustison, J. Wu, P. A. Cook, J. C. Gee, An open source multivariate framework for n-tissue segmentation with evaluation on public data. *Neuroinformatics* **9**, 381-400 (2011).
31. L. Kasper *et al.*, The PhysIO toolbox for modeling physiological noise in fMRI data. *J. Neurosci. Methods* **276**, 56-72 (2017).
32. RCoreTeam, *R: A language and environment for statistical computing* (R Foundation for Statistical Computing, 2021).
33. C. Müller-Axt, C. Eichner, Data from “Data Repository: Mapping the human lateral geniculate nucleus and its cytoarchitectonic subdivisions using quantitative MRI”. Open Science Framework. Available at https://osf.io/tqayf/. Deposited 27 September 2021.
34. C. Müller-Axt *et al.*, Mapping the human lateral geniculate nucleus and its cytoarchitectonic subdivisions using quantitative MRI. *Neuroimage* **244,** 118559 (2021).
35. A. J. Andrews, S. D. Halpern, D. Purves, Correlated size variations in human visual cortex, lateral geniculate nucleus, and optic tract. *J. Neurosci.* **17**, 2859-2868 (1997).
36. C. R. Pernet, Misconceptions in the use of the General Linear Model applied to functional MRI: A tutorial for junior neuro-imagers. *Front. Neurosci.* **8** (2014).
37. K. J. Friston, J. T. Ashburner, S. J. Kiebel, T. E. Nichols, W. D. Penny, *Statistical Parametric Mapping: The analysis of functional brain images* (Academic Press, 2007).
38. C. Chang, J. P. Cunningham, G. H. Glover, Influence of heart rate on the BOLD signal: The cardiac response function. *Neuroimage* **44**, 857-869 (2009).
39. R. M. Birn, M. A. Smith, T. B. Jones, P. A. Bandettini, The respiration response function: The temporal dynamics of fMRI signal fluctuations related to changes in respiration. *Neuroimage* **40**, 644-654 (2008).
40. G. H. Glover, T. Q. Li, D. Ress, Image-based method for retrospective correction of physiological motion effects in fMRI: RETROICOR. *Magn. Reson. Med*. **44**, 162-167 (2000).
